# MicroRNA expression in Epstein-Barr virus-associated post-transplant smooth muscle tumours is related to leiomyomatous phenotype

**DOI:** 10.1186/2045-3329-3-9

**Published:** 2013-07-06

**Authors:** Danny Jonigk, Nicole Izykowski, Lavinia Maegel, Eileen Schormann, Britta Maecker-Kolhoff, Florian Laenger, Hans Kreipe, Kais Hussein

**Affiliations:** 1 Institute of Pathology, Hannover Medical School, Carl-Neuberg-Str. 1, Hannover, D-30625, Germany; 2 Department of Paediatric Haematology and Oncology, Hannover Medical School, Hannover, Germany; 3 Integrated Research and Treatment Center Transplantation (IFB-Tx), Hannover, Germany

**Keywords:** Epstein-Barr virus, EBV, Post-transplant smooth muscle tumour, PTSMT, MicroRNA

## Abstract

Epstein-Barr virus (EBV)-associated post-transplant smooth muscle tumours (PTSMT) are rare complications. In our previous molecular analysis, we have evaluated the expression of regulatory microRNA which are known to be EBV-related (miR-146a and miR-155) but found no deregulation in PTSMT. In this current analysis, we aimed to characterize the expression profiles of several hundred microRNA. Tissue samples from PTSMT and uterine leiomyomas were analysed by quantitative real-time PCR for the expression of 365 mature microRNA. PTSMT and leiomyomas share a highly similar microRNA profile, e.g. strong expression of miR-143/miR-145 cluster and low expression of miR-200c. Among EBV-related microRNA (miR-10b, miR-21, miR-29b, miR-34a, miR-127, miR-146a, miR-155, miR-200b, miR-203 and miR-429) only miR-10b and miR-203 were significantly deregulated. The expression pattern of microRNA in PTSMT is not associated with EBV infection but reflects the leiomyomatous differentiation of the tumour cells.

## Introduction

Epstein-Barr virus (EBV)-associated diseases are often associated with acquired or congenital immunosuppression or immunodeficiency, e.g. bone marrow and solid organ-transplanted patients are at a higher risk. Up to 10% of transplant recipients develop post-transplant lymphoproliferative disorders (PTLD) while EBV-associated post-transplant smooth muscle tumours (PTSMT) are rare complications (<1% of transplant patients) [1,2]. Neoplastic spindle cells in PTSMT express leiomyogenous marker proteins such as smooth muscle actin and desmin, and the majority of tumour cells is positive for EBER. We could previously show that PTSMT differ from conventional leiomyosarcomas by their lack of marked atypia, unusual sites of involvement (>50% in the recipient or donor liver) and defined EBV association [2].

The molecular pathobiology of this rare neoplastic entity is not fully understood and only few experimental analyses have addressed this issue. Ong et al. [3] have analysed cell cycle factors, cytokines and gene promoter methylation in PTSMT and found an activated mTOR/Akt cell cycle pathway by demonstrating phosphorylated mTOR in tumour cells. In our previous analysis, we have evaluated the expression of EBV-associated human genes in PTSMT, including transcription, cell cycle and apoptosis factors and cytokines/cytokine receptors [2]. We found that the transcription factor v-myc myelocytomatosis viral oncogene homolog (avian) (MYC) is significantly upregulated in PTSMT. In addition to mRNA, we have analysed microRNAs which are known to be expressed in an EBV-related fashion (miR-146a and miR-155) but in PTSMT we found low expression levels and no delimitable deregulation. MicroRNAs are non-coding RNA molecules of 20–25 nucleotides in length [4,5]. These small RNA molecules can bind semi-complementarily to the 3′-untranslated region (3′-UTR) of target mRNAs and repress translation or target mRNA for degradation. The microRNA genes can be present as single gene or gene clusters (different microRNA species are encoded on the same chromosome segment). Furthermore, microRNA families represent different microRNA genes with different precursor forms but very similar mature microRNA with no or minor differences in their nucleotide sequence.

Aberrant microRNA expression patterns have been identified in several neoplasms and are known to contribute to the deregulated cell homeostasis in tumour cells, e.g. leiomyomas [6-17]. In this current analysis, we aimed to characterize the expression profiles of several hundred microRNA in PTSMT, in particular regarding an association with smooth muscle phenotype and EBV infection.

## Material and methods

### Tissue specimens

All available PTSMT samples from our tissue archive were evaluated; these PTSMT cases have been characterized earlier [2]. Five EBV^+^ PTSMT samples from four patients, including two tumours from one patient (#4) were analysed (Additional file 1: Table S1). Controls: seven EBV^-^ benign uterine leiomyomas. Formalin-fixed and paraffin-embedded (FFPE) samples were retrieved from the archives of the Institute of Pathology (Hannover Medical School/MHH, Hannover, Germany). The retrospective analysis of the samples has been approved by the local ethics committee of the Hannover Medical School (MHH).

### Laser microdissection of the PTSMT compartment and gene expression analysis

Tissue from FFPE blocks with >90% tumour cells were cut and processed for further PCR analysis. In blocks with <90% aberrant cells, the PTSMT compartments were laser microdissected using a SmartCutPlus-System (MMI, Glattbrugg, Switzerland), as previously described [2].

A set of 365 mature microRNA and corresponding endogenous controls were analysed by quantitative real-time PCR (Pool A, Applied Biosystems, Carlsbad, CA, USA). In brief, cells were digested in proteinase K and RNA was extracted with phenol/chloroform [2,18,19]. Synthesis of cDNA from microRNA, subsequent pre-amplification of cDNA and real-time quantitative PCR with a 7900HT Fast Real-Time PCR system were performed according to the manufacturers’ instructions (Applied Biosystems).

### Data analyses

The sample- and detector-specific evaluation of amplification curves was accomplished with the software RQ Manager 1.2 (Applied Biosystems). C_T_ values established in this manner were converted into ΔC_T_ values and into 2^-ΔCT^ values (normalized to mean of endogenous control genes). Statistical analysis was performed with Prism 5.0 (GraphPad Software, San Diego, California, USA) by applying the Mann–Whitney test for two-group comparison. P values < 0.05 were considered as statistically significant. Cluster analysis was performed with the Qlucore Omics Explorer 2.2 (Qlucore AB, Lund, Sweden).

For target prediction, an open access bioinformatics platform was used (http://www.targetscan.org/).

## Results

### Similar microRNA expression profile in PTSMT and leiomyomas

Cluster analysis of the expression profile of 365 microRNA revealed that PTSMT and leiomyomas share a highly similar profile (Additional file 2: Table S2); cluster analysis could not discriminate between the two tumours.

In PTSMT, only 15/365 microRNA and in leiomyomas 23/365 microRNA showed a mean relative expression level of >1 (Table 1). The five microRNA with the highest expression levels in PTSMT and leiomyomas were the miR-143/miR-145 cluster (both genes are encoded on chromosome region 5q32), miR-24 (two genes on segments 9q22.32 and 19p13.13 encode for the mature miR-24 molecule), let-7b (22q13.31) and miR-21 (17q23.1). In general, most microRNA were expressed at low levels (<1 relative expression level) in PTSMT as well as in leiomyomas, e.g. the leiomyomatous phenotype-associated miR-150 (mean relative level of 0.38 in leiomyomas versus 0.37 in PTSMT), miR-221 (0.09 versus 0.01) and miR-200c (0.00 versus 0.01).

**Table 1:** MicroRNA with a mean relative expression level of >1 (p <0.05*; p <0.01**)

**microRNA**	**PTSMT (mean)**	**Leiomyomas (mean)**
miR-145	36.82	36.01
miR-24	10.63	20.75
miR-143	7.35	8.12
let-7b	2.61	2.79
miR-223	1.98	0.43
miR-342-3p	1.96	1.65
miR-133a	1.65	2.79
miR-19b	1.63	2.23
miR-320	1.49	1.42
miR-21	1.34	5.40
miR-191	1.33	2.38
miR-30c	1.33	1.23
miR-17	1.15	1.20
miR-106a	1.14	1.18
miR-126	1.05	4.74**
miR-222	0.75	7.20**
miR-29a	0.34	2.45*
miR-16	0.68	1.98
miR-214	0.11	1.73**
miR-193b	0.38	1.34*
let-7e	0.19	1.25**
miR-125b	0.01	1.21
miR-199a-3p	0.09	1.06**
miR-100	0.01	1.01**

**Figure 1:**
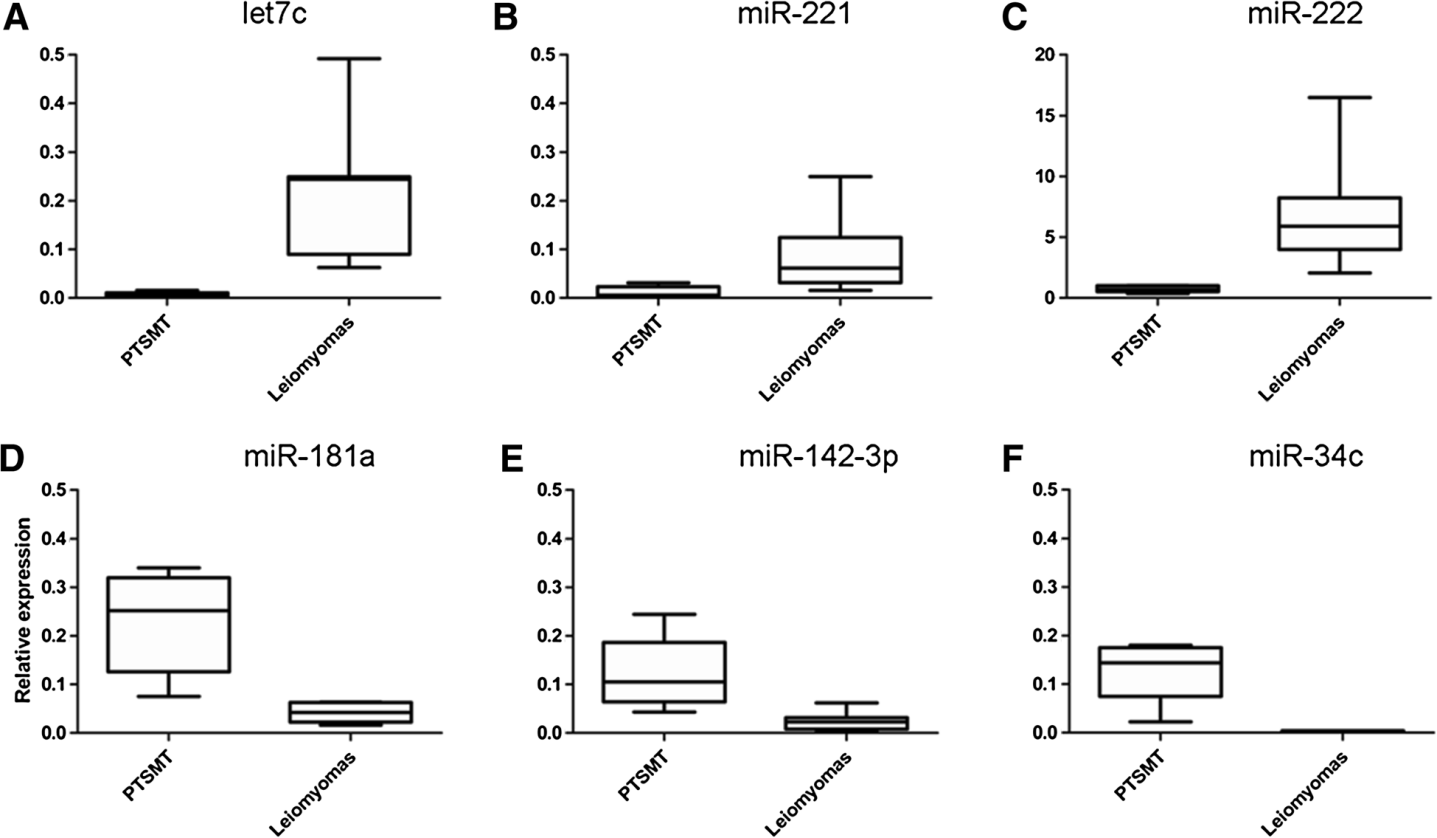
**Representative selection of significantly (p <0.01) de-regulated microRNA expression in PTSMT. A-C)** Examples of down-regulated microRNA. **D-F)** Up-regulated microRNA in PTSMT show relative expression levels below 0.5.

In PTSMT, 59/365 microRNA were significantly deregulated compared with leiomyomas: a set of 51/59 microRNA were down-regulated and 8/59 were up-regulated (Figure 1). However, most of these significances in down-regulated microRNA are a result of very low expression in PTSMT versus low expression in leiomyomas (<1 mean relative expression level), e.g. let-7c (0.22 in leiomyomas versus 0.01 in PTSMT) and miR-221 (0.09 versus 0.01). Furthermore, significantly up-regulated microRNA in PTSMT were a result of very low expression levels in leiomyomas rather than biologically relevant increased expression in PTSMT. In these 8/59 microRNA, the highest relative expression levels were less than 0.5 (miR-181a, miR-34c, miR-142-3p; Figure 1) or even less than 0.05 in PTSMT and leiomyomas (miR-138, miR-181c, miR-190, miR-330-3p/-5p, miR-504).

### Minor association of microRNA expression with EBV infection in PTSMT

The following microRNA are known to be related to EBV infection in solid tumours and haematopoietic malignancies (Additional file 3: Table S3) [20-33]: miR-10b, miR-21, miR-29b, miR-34a, miR-127, miR-146a, miR-155, miR-200b, miR-203 and miR-429. Only two of these microRNA were significantly down-regulated in EBV^+^ PTSMT: miR-10b (mean relative expression of 0.45 in leiomyomas versus 0.01 in PTSMT) and miR-203 (0.01 versus 0.00). Furthermore, in leiomyomas, the level of miR-21 was higher than in PTSMT but the difference did not reach statistical significance (5.40 versus 1.34; p = 0.0876). As could be expected from our previous experiments, miR-146a and miR-155 were not deregulated while the miR-146a-homolog miR-146b was significantly down-regulated in PTSMT (mean 0.23 versus 0.73 in leiomyomas; p = 0.0101). Other EBV-related microRNA (miR-29b, miR-34a, miR-200b and miR-429) were expressed at similarly low levels in both leiomyomas and PTSMT (not significantly deregulated).

## Discussion

### No EBV-related microRNA deregulation in EBV^+^/LMP1^-^ PTSMT

In our first molecular analysis of PTSMT, we analysed the expression of miR-146a and miR-155 because these microRNA are known to be involved in the pathobiology of EBV infection of B cells [2,34,35]. In our current analysis, we could confirm our previous results and found no significant deregulation of these two microRNA using a microarray technique. Furthermore, it is remarkable that several EBV-related microRNA, other than miR-146a and miR-155, are not deregulated in PTSMT. In particular, miR-10b, miR-21, miR-29b, miR-34a and miR-127, which are increased in EBV^+^ nasopharyngeal carcinoma and high grade B cell lymphomas/Burkitt lymphomas [20-33], were expressed at low or very low levels in PTSMT. In non-PTSMT EBV^+^ carcinomas and lymphomas miR-200b, miR-203 and miR-429 are generally expressed at low levels [20-33]. In PTSMT, but also in EBV^-^ leiomyomas, these microRNAs were also expressed at low levels, indicating no specific EBV-related decreased expression in smooth muscle tumours. It has to be taken into account that different cell and tumour types can react in different manners to EBV infection. Furthermore, depending on the latency type, EBV induces expression of different virus proteins, which interfere in the host cell cycle. Latent membrane protein 1 (LMP1) is one of these EBV proteins and it has been shown that LMP1 alone can induce altered microRNA expression in nasopharyngeal carcinomas and lymphomas [20-33]. We and others have found previously that EBV^+^ PTSMT express EBNA2 and EBNA3 while LMP1 expression is weak or not detectable [2,3,36,37]. Thus, despite EBV infection in PTSMT, our finding of no major changes in the microRNA expression profile is likely to be related to lack of LMP1 expression.

### Leiomyomatous phenotype-associated microRNA expression in PTSMT

MicroRNA expression analyses in uterine leiomyomas and leiomyosarcomas were introduced only a few years ago (Additional file 4: Table S4) [6-17]. The majority of studies have evaluated patient-derived leiomyomas as a model disease of neoplastic smooth muscle proliferation. Among different studies and different analytical methods, microRNA expression patterns of patient-derived tumour samples showed a set of microRNA which are recurrently deregulated in comparison to normal uterus wall cells. Similar to PTSMT, decreased expression of miR-150, miR-200c and miR-221 and increased expression of miR-21 and let-7 family members have become particularly evident in leiomyomas [6-17]. It has been demonstrated that mesenchymal cells which differentiate into smooth muscle cells *in vitro* change their microRNA expression patterns, e.g. down-regulation of 13q31.3-clustered miR-17/miR-18a/miR-20a and up-regulation of miR-181a/miR-181c paralogs [16]. Furthermore, in smooth muscle cells, it has been shown that the 5q32-encoded miR-143/miR-145 cluster is co-expressed [38,39]. Both microRNA target a network of factors to promote vascular smooth muscle cell differentiation and repress proliferation [38,39]. We could previously demonstrate high expression of miR-143 and even higher expression of miR-145 in pulmonary vessel wall cells [40]. A similar expression pattern of high miR-143 and higher miR-145 could also be found in PTSMT but also in leiomyomas. Because PTSMT can be found next to vessels (e.g. manifestation in cerebral sinus), it is thought that the aberrant founder cells might be derived from a vessel wall [2,41]. However, due to very similar miR-143/miR-145 expression patterns in PTSMT, uterus wall-derived leiomyomas and pulmonary vessels, the high expression of these two microRNA does not prove a vessel wall origin of PTSMT but reflects the smooth muscle differentiation.

In PTSMT and leiomyomas, many microRNA are expressed at low or very low levels, which makes it likely that protein translation of potential target mRNA types is not inhibited. The problem for target prediction, but simultaneously the hallmark of microRNA/mRNA biology, is the characteristic semi-complementary binding of the seven nucleotides at the 3′-end of the mature microRNA (so-called seed sequence) to corresponding mRNA-nucleotides of the 5′-UTR [4,5]. This semi-complementary binding is sufficient to induce a biological effect, the inhibition of mRNA/protein translation. As a result, one microRNA can bind to several 5′-UTR-mRNA and *vice versa* one 5′-UTR-mRNA can be targeted by several microRNA. In our previous analysis, we have evaluated the expression of several mRNA transcripts in PTSMT and leiomyomas, including MYC, vascular endothelial growth factor A (VEGFA), nuclear factor of kappa light polypeptide gene enhancer in B-cells 1 (NFKB1), tumour protein p53 (TP53), transforming growth factor, beta receptor II (TGFBR2) and transforming growth factor, beta 1 (TGFB1) [2].

Among several EBV-associated human factors, we found only MYC to be significantly increased in PTSMT [2]. The mRNA of this transcription factor can be regulated by miR-150, miR-143 and miR-145 [42,43] but no PTSMT-specific inverse correlation was found.

VEGFA can be negatively regulated by miR-200c and other microRNA. In leiomyomatous cell lines, miR-200c interaction with VEGFA has been shown [7] and accordingly, in leiomyomas as well as in PTSMT, very low levels of miR-200c correlate with increased levels of VEGFA [2].

In many different tumour types, miR-21 is aberrantly expressed, because this microRNA can target several signal networks, either directly by binding to different types of mRNA from similar signal cascades or indirectly via deregulation of factors down/up-stream to the factors directly suppressed by miR-21 [44,45]. In particular, miR-21 is a negative regulator of TP53 signalling and simultaneously a promoter of NFKB1 signalling [44,45]. Increased expression of miR-21 has been previously demonstrated in leiomyomas [6,10,15,16] which we could confirm in our analysis. In smooth muscle cells, miR-21 is involved in regulation of apoptosis and TGFBR2/TGFB1 signalling [6,10,13]. TGFBR2-3′UTR has an miR-21 binding site and can therefore directly be regulated by miR-21 in smooth muscle cells [10]. *In vitro* studies also suggested an indirect regulatory interaction between miR-21 and TGFB1; of note, TGFB1 is not a direct target of miR-21 [10]. Furthermore, inhibition of miR-21 expression in smooth muscle cells indirectly increases caspase 3 and caspase 7 activity *in vitro*; both caspases have no miR-21 binding site [6,13]. The miR-21 expression was lower in PTSMT than leiomyomas but the difference was not significant. In addition, in our previous analysis we found no differences in the expression of miR-21-related NFKB1, TP53, TGFBR2 or TGFB1 between PTSMT and leiomyomas [2]. Therefore, our *in situ*-derived results do not reveal a PTSMT-specific deregulated miR-21 signal cascade, but an expression pattern related to smooth muscle phenotype.

Members of the let-7 family are increased in leiomyomas [15] and smooth muscle cell lines, e.g. let-7b [13,16]. We found that in PTSMT and leiomyomas, the strongest expressed let-7 paralog was let-7b. In leiomyomas and leiomyosarcomas, let-7c shows an inverse correlation with its target mRNA high mobility group AT-hook 2 (HMGA2) [11,17]. Both genes, let-7c and HMGA2, are expressed in association with size of leiomyomas [11], while in PTSMT, irrespective of the size, let-7c was almost absent.

In summary, in addition to leiomyomas and leiomyosarcomas, PTSMT is the third smooth muscle tumour type in which the microRNA expression profile could be evaluated. The expression pattern of microRNA in PTSMT is not associated with EBV infection (presumably due to lack of strong LMP1 expression) but reflects the leiomyomatous differentiation of the tumour cells.

## Competing interests

The authors declare that they have no competing interests.

## Authors’ contributions

Conception of analysis (KH, DJ), histomorphology (DJ, KH, HK, FL), molecular analysis (LM, NI, ES, KH, DJ), data collection, analysis of data and manuscript preparation (DJ, FL, LM, NI, ES, BMK, HK, KH). All authors read and approved the final manuscript.

## Supplementary Material

Additional file 1: Table S1 Characteristics of patients with PTSMT. Click here for file

Additional file 2: Table S2 MicroRNA expression in PTSMT in comparison to previously published data on leiomyomas (LM) and leiomyosarcomas (LMS). See Additional file 4: Table S4 for details on published data. Click here for file

Additional file 3: Table S3 MicroRNA expression in association with EBV. Click here for file

Additional file 4: Table S4 Summary of microRNA expression data in leiomyomas and leiomyosarcomas. Click here for file
