# Supplementary material for: MicroRNA expression in Epstein-Barr virus-associated post-transplant smooth muscle tumours is related to leiomyomatous phenotype
Source: Clin Sarcoma Res. 2013 Jul 6;3:9. doi: 10.1186/2045-3329-3-9 (PMC3706214; doi:10.1186/2045-3329-3-9)
Supplement: Additional file 1: Table S1 — Characteristics of patients with PTSMT. [file 2045-3329-3-9-S1.doc]

**Additional file 1: Table S1. Characteristics of patients with PTSMT.**

|  | **Gender, age** | **Transplant organ** | **Tumour manifestation and localization** |
| --- | --- | --- | --- |
| **#1** | ♀, 6 years | Liver | +16 months after transplantation, EBV^+^ PTSMT (liver) |
| **#2** | ♀, 7 years | Liver | +68 months after transplantation, EBV^+^ PTSMT (bronchus) |
| **#3** | ♀, 15 years | Heart | +28 months after transplantation, EBV^+^ PTSMT (colon) |
| **#4** | ♀, 13 years  (Fanconi anaemia) | Bone marrow | +52 months after transplantation, two EBV^+^ PTSMT (cerebral sinus/#4-1 and spleen/#4-2) |
| **#5-#11** | 7 ♀, Median 39 (32-55) | Kidney | +82 months after transplantation, EBV^-^ leiomyomas (uterus), 55-year-old patient |
|  |  | No transplantation | EBV^-^ leiomyomas (uterus), six patients |
