# Supplementary material for: MicroRNA expression in Epstein-Barr virus-associated post-transplant smooth muscle tumours is related to leiomyomatous phenotype
Source: Clin Sarcoma Res. 2013 Jul 6;3:9. doi: 10.1186/2045-3329-3-9 (PMC3706214; doi:10.1186/2045-3329-3-9)
Supplement: Additional file 2: Table S2 — MicroRNA expression in PTSMT in comparison to previously published data on leiomyomas (LM) and leiomyosarcomas (LMS). See Additional file 4: Table S4 for details on published data. [file 2045-3329-3-9-S2.doc]

**Additional file 2: Table S2. MicroRNA expression in PTSMT in comparison to previously published data on leiomyomas (LM) and leiomyosarcomas (LMS). See Additional file 4: Table S4 for details on published data.**

| **microRNA** | **Previously published data** | **PTSMT**  **(mean)** | **Leiomyomas**  **(mean)** | **Significance** |
| --- | --- | --- | --- | --- |
| let-7a | Up in LM | 0.11 | 0.24 | p = 0.0303 |
| let-7b | Up in LM | 2.61 | 2.79 | n.s. |
| let-7c | Up in LM | 0.01 | 0.22 | p = 0.0025 |
| let-7d | Up in LM | 0.05 | 0.10 | n.s. |
| let-7e | Up in LM | 0.19 | 1.25 | p = 0.0025 |
| let-7f | Up in LM | 0.01 | 0.01 | n.s. |
| let-7g | Up in LM | 0.09 | 0.22 | p = 0.0480 |
| miR-15b | Up in LMS | 0.04 (miR-15a 0.02) | 0.07  (miR-15a 0.03) | n.s. |
| miR-21 | Up in LM | 1.34 | 5.40 | n.s. |
| miR-23b | Up in LM | 0.03 (miR-23a 0.04) | 0.11 (miR-23a 0.06) | p = 0.0025 (n.s.) |
| miR-27a | Up in LM | 0.14 (miR-27b 0.14) | 0.18 (miR-27b 0.36) | n.s. (p = 0.0190) |
| miR-29b | Down in LM | 0.00 (miR-29a 0.34; miR-29c 0.26) | 0.01 (miR-29a 2.45; miR-29c 0.36) | n.s. (p = 0.0101) |
| miR-30a | Up in LM | Not analysed | Not analysed |  |
| miR-30b | Down in LM | 0.50 | 0.56 | n.s. |
| miR-30c | Down in LM | 1.33 | 1.23 | n.s. |
| miR-32 | Down in LM | 0.00 | 0.00 | n.s. |
| miR-34a | Up in LM | 0.27 (miR-34c 0.13) | 0.46 (miR-34c 0.00) | n.s. (p = 0.0025) |
| miR-125b | Up in LM | 0.01 (miR-125a-3p 0.00; miR-125a-5p 0.02) | 1.21 (miR-125a-3p 0.00; miR-125a-5p 0.03) | p = 0.0025 (n.s.; n.s.) |
| miR-130b | Up in LMS | 0.01 (miR-130a 0.03) | 0.01 (miR-130a 0.08) | n.s. |
| miR-135 | Up in LM | miR-135a 0.00; miR-135b 0.29 | miR-135a 0.00; miR-135b 0.05 | n.s. |
| miR-139 | Down in LM  Down in LMS | miR-139-3p 0.01; miR-139-5p 0.02 | miR-139-3p 0.00; miR-139-5p 0.00 | n.s. |
| miR-150 | Down in LM  Down in LMS | 0.37 | 0.38 | n.s. |
| miR-197 | Down in LM | 0.07 | 0.04 | n.s. |
| miR-200c | Down in LM | 0.01 (miR-200a 0.00; miR-200b 0.00) | 0.00 (miR-200a 0.00; miR-200b 0.00) | n.s. (n.s.; n.s.) |
| miR-212 | Down in LM | 0.01 | 0.02 | n.s. |
| miR-217 | Down in LM  Down in LM | Not detectable | 0.00 |  |
| miR-221 | Not detectable in LM, detectable  in LMS | 0.01 | 0.09 | p = 0.0051 |
| miR-301 | Not detectable in LM, detectable  in LMS | 0.02 | 0.01 | n.s. |
| miR-323 | Up in LM | miR-323-3p 0.02 | miR-323-3p 0.00 | n.s. |
| miR-329 | Down in LMS | 0.00 | 0.00 | n.s. |
| miR-363 | Up in LM | 0.00 | 0.00 | n.s. |
| miR-370 | Up in LMS | 0.03 | 0.02 | n.s. |
| miR-376a | Not detectable in LM, detectable  in LMS | 0.02 (miR-376b 0.00; miR-376c 0.07) | 0.04 (miR-376b 0.00; miR-376c 0.20) | n.s. |
| miR-451 | Down in LM | 0.01 | 0.01 | n.s. |
| miR-490 | Up in LM  Up in LMS | 0.00 | 0.00 | n.s. |
| miR-495 | Down in LM  Down in LMS | 0.01 | 0.00 | n.s. |
| miR-508 | Down in LMS | Not detectable | 0.00 |  |
| miR-590 | Down in LM | miR-590-5p 0.01 | miR-590-5p 0.02 | p = 0.0303 |
