# Supplementary material for: MicroRNA expression in Epstein-Barr virus-associated post-transplant smooth muscle tumours is related to leiomyomatous phenotype
Source: Clin Sarcoma Res. 2013 Jul 6;3:9. doi: 10.1186/2045-3329-3-9 (PMC3706214; doi:10.1186/2045-3329-3-9)
Supplement: Additional file 3: Table S3 — MicroRNA expression in association with EBV. [file 2045-3329-3-9-S3.doc]

**Additional file 3: Table S3. MicroRNA expression in association with EBV.**

| **microRNA** | **Previously published data (reference)** | **PTSMT**  **(mean)** | **Leiomyomas**  **(mean)** |
| --- | --- | --- | --- |
| miR-10b | LMP1-associated up in nasopharyngeal carcinoma cell line (26) | 0.01 | 0.45 |
| miR-21 | EBNA2-associated up in B cell line (21) | 1.34 | 5.40 |
| miR-29b | LMP1-associated up in B cell line (31) | 0.00 | 0.01 |
| miR-34a | LMP1-associated up in B cell line (22) | 0.27 | 0.46 |
| miR-127 | EBNA1-associated up in B cell line (20) | 0.09 | 0.03 |
| miR-146a | EBNA1-associated down in B cell line (21)  LMP1- associated up in B cell line (33) | 0.62 | 0.32 |
| miR-155 | LMP1/LMP2A-associated up in nasopharyngeal carcinoma cell line and B cell line (24,33) | 0.11 | 0.13 |
| miR-200b | Negative correlation with ZEB1/ZEB2 and positive correlation with lytic phase in nasopharyngeal carcinoma cell line, gastric carcinoma cell line and B cell line (27,28,29) | 0.00 | 0.00 |
| miR-203 | LMP1-associated down in nasopharyngeal carcinoma cell line (23) | 0.00 | 0.01 |
| miR-429 | Negative correlation with ZEB1/ZEB2 and positive correlation with lytic phase in nasopharyngeal cell line carcinoma and B cell line (27,28) | 0.00 | 0.00 |
