# Supplementary material for: MicroRNA expression in Epstein-Barr virus-associated post-transplant smooth muscle tumours is related to leiomyomatous phenotype
Source: Clin Sarcoma Res. 2013 Jul 6;3:9. doi: 10.1186/2045-3329-3-9 (PMC3706214; doi:10.1186/2045-3329-3-9)
Supplement: Additional file 4: Table S4 — Summary of microRNA expression data in leiomyomas and leiomyosarcomas. [file 2045-3329-3-9-S4.doc]

**Additional file 4: Table S4. Summary of microRNA expression data in leiomyomas and leiomyosarcomas.**

| **Tumour type, number of cases** | **Analytical method for microRNA analysis** | **Main findings** | **Reference** |
| --- | --- | --- | --- |
| Uterine leiomyomas (11 tumours, patient samples), control: uterine myometrium | qPCR | *In situ*: Elevation of miR-21 in leiomyomas.  *In vitro*: Increased miR-21 in UTLM but no miR-21/PDCD-4 regulation; inhibition of miR-21 results in increased CASP3 cleavage and EF2 expression (indirect miR-21 effect) | Fitzgerald et al., 2012 (6) |
| Uterine leiomyomas (23 tumours patient samples), control: uterine myometrium | qPCR | *In situ*: Increased miR-21 in leiomyomas, drug-associated increase of miR-21 in leiomyomas  *In vitro*: miR-21 regulation of PTEN, PDCD4, E2F1 and TGFBR2 (cell lines MSMC, LSMC, t-LSMC and SKLMS1)  *In vitro*: inhibition of miR-21 results in increased CASP3/CASP7 activity (indirect miR-21 effect) and cell viability (LSMC, t-LSMC and SKLMS1 but not MSMC) | Pan et al., 2010 (10) |
| Uterine leiomyomas (7 tumours patient samples), control: uterine myometrium | microRNA microarray, qPCR | *In situ*: lower expression of miR-20a, miR-21, miR-26a, miR-18a, miR-206, miR-181a and miR-142-5p in leiomyomas from African Americans than Caucasians  *In vitro*: >50 deregulated microRNA (cell lines MSMC, t-LSMC, LSMC and SKLMS1), Top 5 up-regulated: miR-145, miR-125, let-7 family, miR-23, miR-143.  *In vitro*: ovarian steroid-associated down-regulation of miR-20a, miR-21 and miR-26a. | Pan et al., 2008 (13) |
| Uterine leiomyomas (55 tumours, patient samples), control: uterine myometrium | microRNA microarray, qPCR, Northern Blot | *In situ*: 45 deregulated microRNA. Top 5 up-regulated: let-7 family, miR-21, miR-23b, miR-27a, miR-30a. Top 5 down-regulated: miR-29b, miR-32, miR-144, miR-197, miR-212. | Wang et al., 2007 (15) |
| Uterine leiomyomas (15 tumours patient samples), control: uterine myometrium | microRNA microarray, qPCR | *In situ*: 81 deregulated microRNA. Top 5 up-regulated (microarray): miR-542-3p, miR-377, miR-582, miR-376b, miR-493-5p; (qPCR validation: miR-21, miR-34a, miR-125b, miR-323). Top 5 down-regulated (microarray): miR-542-5p, miR-642, miR-150, miR-203, miR-139; (qPCR validation: miR-139). | Marsh et al., 2008 (14) |
| Uterine leiomyomas (76 tumours patient samples), control: uterine myometrium | qPCR | *In situ*: Decreased miR-200c in leiomyomas (p < 0.05); lower levels in leiomyomas from African Americans than Caucasians.  *In vitro*: miR-200c/TIMP2 and miR-200c/FBLN5 regulation in MCMC, LSMC, SKLMS1 and miR-200c/VEGFA in SKLMS1 | Chuang et al., 2012 (7) |
| Uterine leiomyomas  (8 tumours patient samples) | Based on data from previous study (Wang et al., 2007) | *In situ*: Deletion of 1p36.33-p36.23 can be associated with decreased 1p36-encoded miR-200a/miR-200b | Zavadil et al., 2010 (9) |
| Uterine leiomyomas, control: uterine myometrium | High-throughput sequencing | *In situ*: >50 deregulated microRNA. Top 5 up-regulated: miR-363, miR-490, miR-137, miR-543y, miR-135b. Top 5 down-regulated: miR-217, miR-4792, miR-590, miR-451b, miR-451 | Georgieva et al., 2012 (Abstract) (8) |
| Uterine leiomyomas (150 tumours, patient samples), control: uterine myometrium | qPCR, microRNA *in situ* Hybridization | *In situ*: Increased let-7/decreased HMGA2 in small leiomyomas (<3 cm) and decreased let-7/increased HMGA2 in large leiomyomas (>7 cm).  *In vitro*: let-7c regulation of HMGA2 is associated with an antiproliferative effect (cell lines PC3 and LNCAP) | Peng et al., 2008 (11) |
| Uterine leiomyosarcomas (35 tumours, patient samples), control: uterine myometrium | qPCR, microRNA *in situ* Hybridization | *In situ*: Inverse correlation of miR-7/HMGA2  In vitro: miR-7c regulation of HMGA2 is associated with an antiproliferative effect (cell lines SKLMS1, SKUT1 and SKUT1b) | Shi et al., 2009 (17) |
| Benign metastasizing leiomyomas of the lung  (10 tumours, patient samples), control: 15 leiomyosarcomas, 8 leiomyomas | microRNA *in situ* Hybridization | *In situ*: MiR-221, miR-301 and miR-376a were not detectable in benign metastasizing and leiomyomas of the lung (n total = 0/18).  MiR-221 (n = 13/15), miR-301 (n = 2/15) and miR-376a (n 2/15) were detectable in leiomyosarcomas | Nuovo et Schmittgen, 2008 (12) |
| Uterine leiomyomas (10 tumours, patient samples), uterine leiomyosarcomas (10 tumours, patient samples), control: uterine myometrium | microRNA microarray, qPCR | *In situ* (leiomyosarcomas versus myometrium): 72 deregulated microRNA. Top 5 up-regulated: miR-490, miR-630, miR-130b, miR-15b, miR-370. Top 5 down-regulated: miR-150, miR-508, miR-495, miR-139, miR-329.  *In situ* (leiomyomas versus myometrium): <10 deregulated microRNA. Up-regulated: miR-34a and miR-21. Down-regulated: miR-150, miR-30b/c, miR-495.  *In vitro*: 30 deregulated microRNA during smooth muscle differentiation of mesenchymal cells. Top 5 up-regulated: miR-181c/a, miR-204, miR-34a/b, miR-373*, miR-498. Top 5 down-regulated: miR-7, miR-224, miR-18a, miR-138, miR-17-5p | Danielson et al., 2010 (16) |
